# CRISPR-Cas10-Assisted Structural Modification of Staphylococcal *Kayvirus* for Imaging and Biosensing Applications

**DOI:** 10.1021/acssynbio.5c00387

**Published:** 2025-07-28

**Authors:** Hana Šimečková, Pavol Bárdy, Lucie Kuntová, Eliška Macháčová, Tibor Botka, Ján Bíňovský, Josef Houser, Zdeněk Farka, Pavel Plevka, Roman Pantůček, Ivana Mašlaňová

**Affiliations:** a Department of Experimental Biology, Faculty of Science, Masaryk University, Brno 611 37, Czech Republic; b Department of Chemistry, York Structural Biology Laboratory, University of York, Heslington, York YO10 5DD, United Kingdom; c Department of Biochemistry, Faculty of Science, 37748 Masaryk University, Brno 625 00, Czech Republic; d Central European Institute of Technology, Masaryk University, Brno 625 00, Czech Republic; e National Centre for Biomolecular Research, Faculty of Science, 37748 Masaryk University, Brno 625 00, Czech Republic

**Keywords:** Bacteriophage, CRISPR-Cas10, *Staphylococcus aureus*, Biosensing Techniques, Poly histidine Tag, *Herelleviridae*

## Abstract

Recent advances in genome editing techniques based on CRISPR-Cas have opened up new possibilities in bacteriophage engineering and, thus, enabled key developments in medicine, nanotechnology, and synthetic biology. Although staphylococcal phage genomes have already been edited, the modification of their structural proteins has not yet been reported. Here, the structure of *Staphylococcus* phage 812h1 of the *Kayvirus* genus was modified by inserting a poly histidine tag into an exposed loop of the tail sheath protein. A two-strain editing strategy was applied, utilizing homologous recombination followed by CRISPR-Cas10-assisted counter-selection of the recombinant phages. The His-tagged phage particles can be recognized by specific antibodies, enabling the modified bacteriophages to be employed in numerous techniques. The attachment of the engineered phage to bacteria was visualized by fluorescence microscopy, and its functionality was confirmed using biolayer interferometry biosensing, enzyme-linked immunosorbent assay, and flow cytometry, demonstrating that the genetic modification did not impair its biological activity.

## Graphical abstract

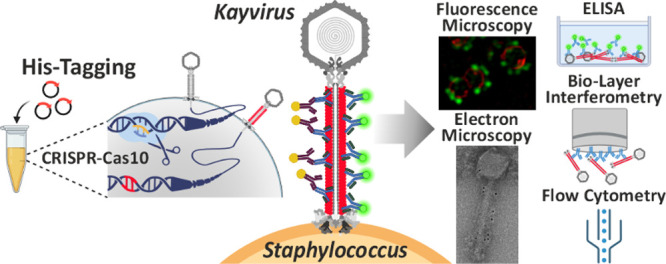

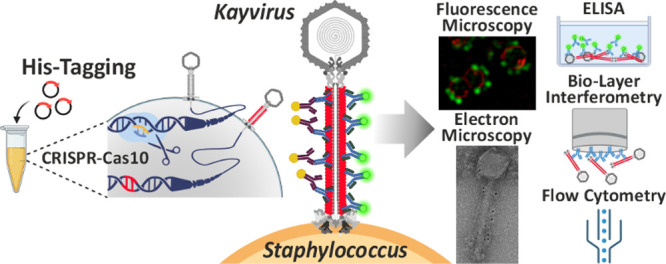

## Introduction

Bacteriophages, natural predators of bacteria, are integral components of a wide range of ecosystems and profoundly influence bacterial evolution.^1^ Fundamental phage research and studies of its applications in medicine, the food industry, and biotechnology are being intensively conducted worldwide. Technological advances in genetic engineering open up new possibilities for improving phage properties, and modified bacteriophages are powerful tools in current science.^2–4^

Phage particles are complex viral DNA–protein assemblies with diverse biological functions and effects on target cells. Genetic modifications of phages focus on extending their host range,^5,6^ increasing their antibacterial efficacy,^7,8^ and the development of reporter phages for pharmaceutical research and biotechnology applications.^9^ Modified phage particles can also act as selective bioreceptors in various detection tools,^10^ deliver a programmed CRISPR-Cas system,^11–13^ or serve as antibacterial drones.^14,15^ However, the efficient genome editing of lytic bacteriophages, which do not integrate DNA into the bacterial chromosome, poses several challenges due to their rapid replication cycle,^16^ degradation of the host chromosome early in infection,^17^ or compartmentalization of their genome from the rest of the cell.^18^

An effective genome-editing strategy that has been successfully implemented in recent years is CRISPR-assisted genome editing. This approach is based on homologous recombination followed by the counter-selection of rare recombinants using the CRISPR-Cas machinery. It has enabled the successful modification of various phages targeting *Escherichia coli*,^19–21^ *Streptococcus thermophilus*,^22^ *Vibrio cholerae*,^23^ *Lactococcus lactis*,^24^ *Klebsiella pneumoniae*,^25^ *Pseudomonas aeruginosa*,^26^ and *Listeria monocytogenes*.^27^

The CRISPR-Cas10 (Type III-A) system to counter-selection of recombinant staphylococcal phages was also applied to the genome editing of *Staphylococcus aureus* kayviruses from the family *Herelleviridae*.^28,29^ However, this was performed as a proof-of-concept study, introducing simple nucleotide substitutions into the gene for the phage DNA polymerase, resulting in silent mutations. Structural studies of kayviruses,^30^ the availability of genomes in public databases, the integration of proteomic data into this framework,^31^ and new insights into phage-host interactions obtained from transcriptomic studies^32^ bring possibilities for more challenging genome editing of this phage group.

Here, we present structural modification of the highly virulent and polyvalent *Staphylococcus* phage 812h1 ^33^ of the *Kayvirus* genus using a modified CRISPR-Cas10-assisted editing and selection strategy. Kayviruses are widely employed in phage therapeutic preparations and are generally considered safe for clinical application.^34,35^ We incorporated a multifunctional poly histidine tag, broadly employed in biological applications, into the tail sheath protein (TSP). The introduced His-tag enabled biosensing detection, ultraresolution visualization using fluorescence and electron microscopy, and the study of phage-host interactions. This demonstrates that the modified phage can be broadly utilized as a model viral particle for monitoring and diagnostic applications in various sample matrices.

## Results and Discussion

The lytic staphylococcal bacteriophage 812h1 with a broad host range, previously derived from phage 812,^33^ was chosen for structural modifications. The virion of phage 812 consists of an isometric head, a long tail with a contractile sheath, and a double-layered baseplate.^30^ The tail consists of tail sheath proteins (TSPs) arranged in hexamer discs surrounding the central tail tube channel (Figure 1CD). Based on the TSP structure (PDB: 5LI2),^30^ an unstructured exposed loop on the surface of TSP (Figure 1BCD, Figure S1) was selected as a target site for introducing modifications. A DNA sequence encoding a string of six histidine residues (6× His) was designed for insertion into the TSP gene (Figure 1A) between the codons for alanine (Ala272) and glutamic acid (Glu273) of the TSP (Figure 1B) using CRISPR-Cas10-assisted phage genome editing derived from the system developed by Bari et al.^28,29^

**Figure 1:**
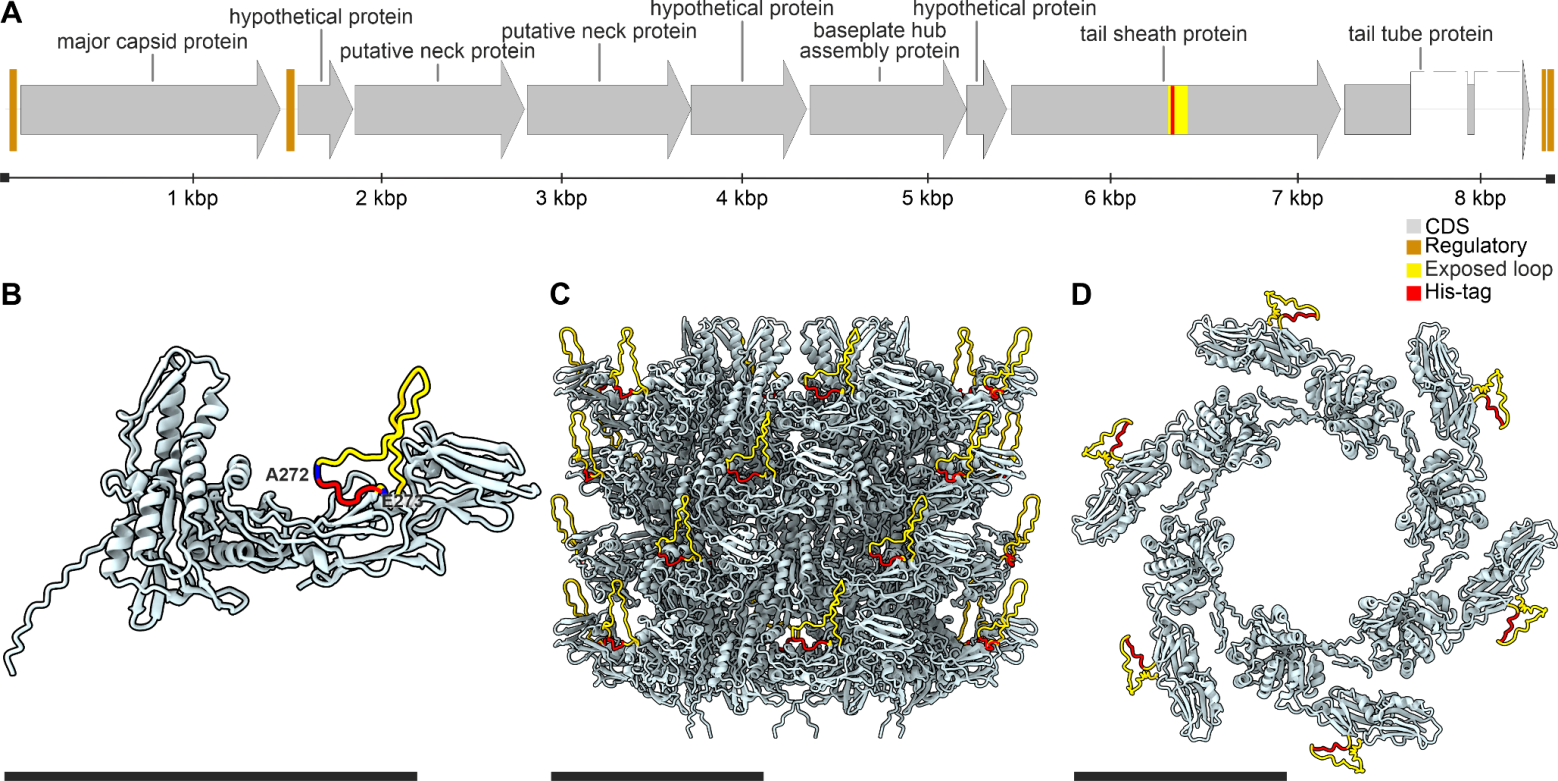
Scheme depicting the introduction of a poly histidine tag into the phage 812h1 tail sheath protein. (A) Part of the 812h1 phage head–tail structural genome module (GenBank: MH844529.1, sequence region: 51781 – 60096) showing the proposed insertion of a His-tag-encoding sequence (red) into the sequence encoding the exposed loop (yellow) within the TSP (GenBank: AZB50028.1, region 271 – 297). (B) Predicted structure of the modified TSP with an exposed loop (yellow) and inserted His-tag (red). The delimiting residues of the insertion Ala272 and Glu273 are highlighted in blue. (C) Modified TSPs with exposed loops (yellow) and inserted His-tags (red), modeled based on the structure of the four tail sheath protein hexamer discs (PDB: 9F04).^36^ Side view of the tail sheath is shown. (D) Top view of a modeled single tail sheath protein hexamer disc shown in panel C. Scale bars are 10 nm (black).

The wild-type phage genome was genetically modified by homologous recombination using editing strain *S. aureus* HR_HisTSP (Figure 2A) derived from laboratory strain *S. aureus* RN4220. The strain contained plasmid pCN-EF2132tet (pCN-EF), which encodes enterococcal recombinase EF2132 for increasing recombination efficiency^37^ and the compatible vector pHR_812HisTSP carrying the template for the homologous recombination (Table 1). The editing template contained an 18-nucleotide *his*-tag (6× CAT) flanked by two 520-bp homologous arms that target the editing site within the exposed loop of the *tsp* gene, between the triplets GCA and GAA (GenBank: MH844529.1, positions 57988 and 57991, respectively; Figure 1A). The phage progeny resulting from propagation of the high-titer phage lysate (10^9^ PFU/mL) on the editing strain was counter-selected using the *S. aureus* CRISPR spc+ selection strain, which harbors the effector vector p*crispr-cas/Δcas1Δcas2* ^29^ (abbreviated pCas10) encoding the endonuclease complex Cas10-Csm^28^ and the vector pCN_gRNAspc+ carrying the CRISPR-Cas10 gRNA scaffold with a spacer that targets only the unmodified *tsp* gene (Figure 2B, Table 1). The *S. aureus* CRISPR spc- strain lacking the specific spacer in the gRNA was used as a control (Table 1).

**Figure 2:**
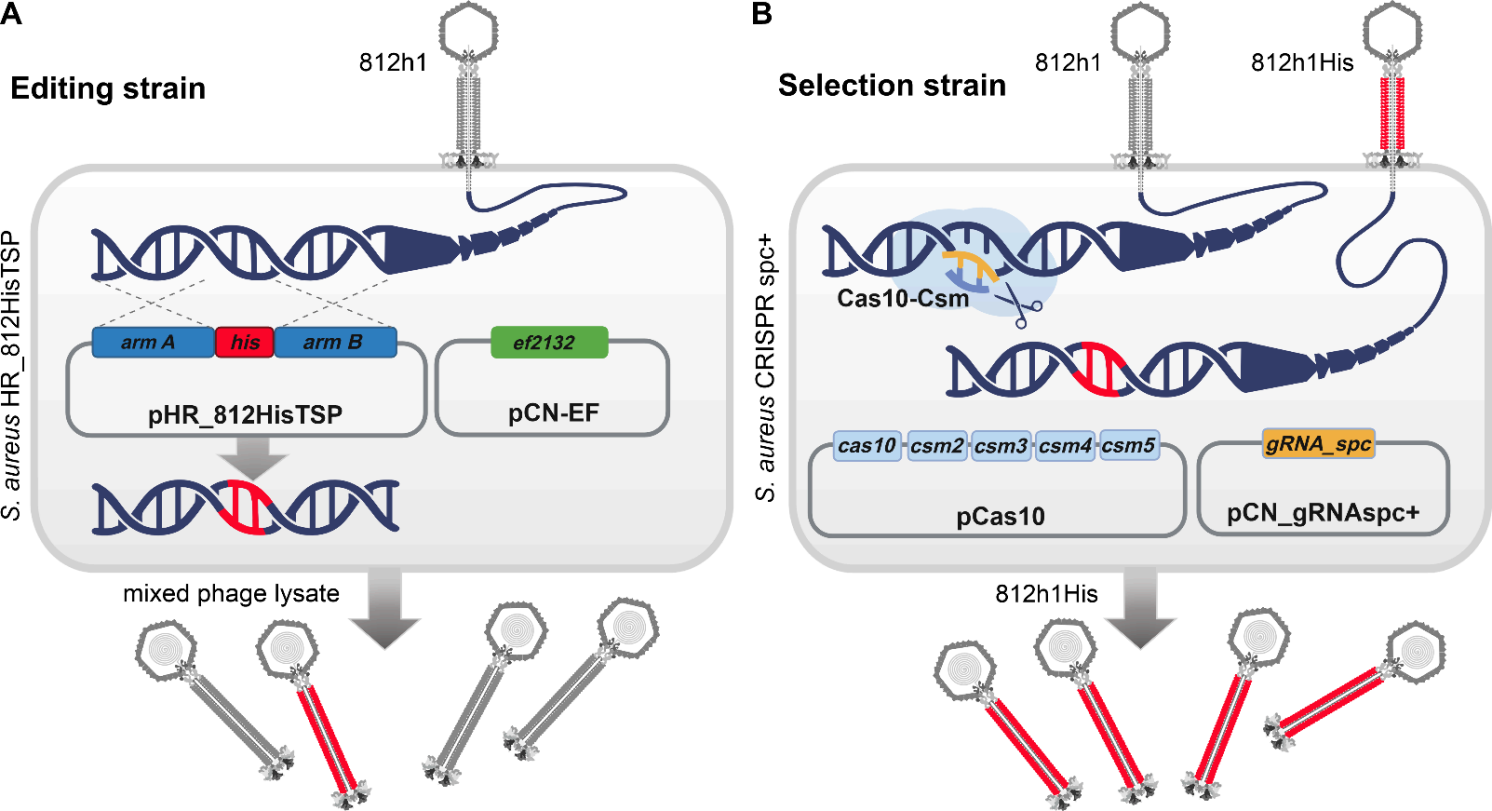
Two-strain CRISPR-Cas10-based editing strategy. (A) Genome editing of phage 812h1. The *S. aureus* HR_812HisTSP editing strain carrying the pHR_812HisTSP and pCN-EF vectors mediates the insertion of a *his*-tag sequence into the gene for the tail sheath protein. After homologous recombination facilitated by recombinase EF2132 between arms A and B, a mixed phage progeny is produced, containing a majority of the wild-type phages (gray) and recombinant phages (red) bearing a His-tag within the tail sheath. (B) Selection of recombinant phages. The *S. aureus* CRISPR spc+ selection strain carrying pCas10 and pCN_gRNAspc+ mediates the recognition and degradation of unedited DNA of phage 812h1 (gray). The Cas10-Csm effector complex is composed of five *cas-csm* genes and is guided to its target by a gRNA with a specific spacer (orange). Recombinant phage 812h1His with an insertion in the genome escapes CRISPR recognition and produces uniform recombinant phage progeny (red).

**Table 1:** Plasmid Vectors Used in This Study

Vector	Length (bp)	Selection marker^a^	Purpose	Reference
pCas10 = p*crispr-cas/Δcas1Δcas2*	10,910	cmp^R^	A component of CRISPR-Cas10 counter-selection that carries genes for the Cas10-Csm effector complex, ensuring the degradation of the targeted DNA.	^29^
pCN_gRNAspc-	6,526	ery^R^	A component of CRISPR-Cas10 counter-selection that carries a gRNA scaffold without any spacer that targets phage 812h1.	This work
pCN_gRNAspc+	6,561	ery^R^	A component of CRISPR-Cas10 counter-selection that carries a gRNA with the spacer that targets the *tsp* gene of phage 812h1.	This work
pCN-EF= pCN-EF2132tet	7,266	cmp^R^	This vector carries the gene for an enterococcal recombinase EF2132.	^37^
pHR_812HisTSP	6,267	ery^R^	A vector complementary to pCN-EF that carries the editing segment HR_812HisTSP for homologous recombination.	This work
pCas9counter	9,533	ery^R^	This vector backbone was used to construct pHR_812HisTSP, compatible with pCN-EF.	^37^
pCN51	6,430	ery^R^	This vector was used to construct the vectors pCN_gRNAspc+ and pCN_gRNAspc-.	^45^

a Legend: cmp^R^, resistance to chloramphenicol (25 μg/mL); ery^R^, resistance to erythromycin (10 μg/mL).

The ability of modified phages to escape CRISPR-Cas-mediated selection was tested in the *S. aureus* CRISPR spc+ selection strain by using a spot plaque assay (Figure 3A). At a concentration of 1 × 10^9^ PFU/mL, phage 812h1 did not form any plaques on *S. aureus* CRISPR spc+, whereas it propagated unimpeded on *S. aureus* CRISPR spc- (Figure 3B). After the editing step, the titer was 1 × 10^9^ PFU/mL on the *S. aureus* CRISPR spc- strain, while the titer on the *S. aureus* CRISPR spc+ strain was (0.9–1.7) × 10^6^ PFU/mL, indicating the emergence of recombinant 812h1His phages with 0.1% recombination efficiency (Figure 3AB). After four passages on the *S. aureus* CRISPR spc+ strain, a homogeneous 812h1His phage progeny was obtained with an almost identical titer on both CRISPR spc+ and CRISPR spc- strains (Figure 3AB). The stability of the 812h1His phage was confirmed by repeated propagation on *S. aureus* RN4220 spc-, with no change in efficiency of plating on *S. aureus* RN4220 spc- and *S. aureus* RN4220 spc+ strains. The modified 812h1His phages retained their infectivity and demonstrated the ability to eradicate *S. aureus* culture in a turbidity assay (Figure 3CD).

**Figure 3:**
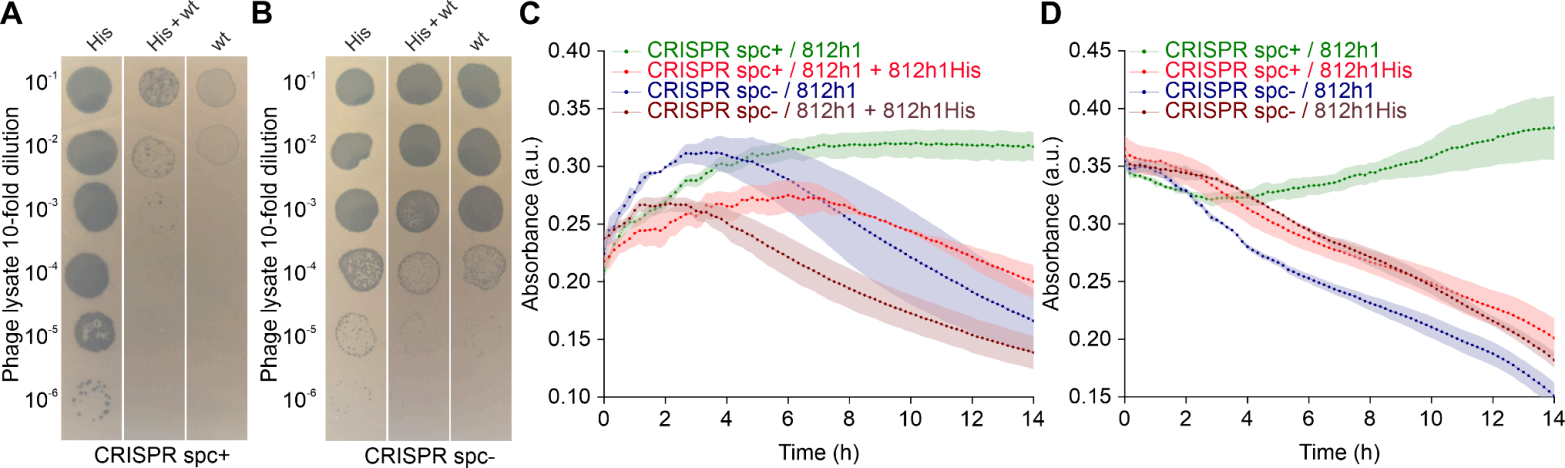
Efficiency of *S. aureus* CRISPR-Cas10 selection system. (A) Double-layer agar overlay plaque spot assay showing the sensitivity of the *S. aureus* CRISPR spc+ strain to the 812h1His (His) phage, mixed phage lysate (His + wt), and phage 812h1 (wt). Wild-type phage 812h1 does not propagate efficiently on the *S. aureus* CRISPR spc+ strain. (B) A plaque spot assay showing the sensitivity of the *S. aureus* CRISPR spc- strain to the 812h1His phage, mixed phage lysate, and phage 812h1. (C) Turbidity assay showing the sensitivity of the *S. aureus* CRISPR spc+ selection strain to mixed phage progeny comprising 812h1 and 812h1His phages. Wild-type phages 812h1 are recognized by the Cas10 effector complex and do not lyse the *S. aureus* CRISPR spc+ selection strain (green). The control *S. aureus* CRISPR spc- strain without a specific spacer is sensitive to 812h1 phages (blue). The mixed phage lysate, which contains a population of 812h1His phages, can lyse both the selection strain (red) and the strain without the specific spacer (brown). (D) Turbidity assay showing the sensitivity of the *S. aureus* CRISPR spc+ selection strain to wild-type phages 812h1 and phage 812h1His. The modified 812h1His phages overcame the CRISPR-Cas10 immune system and efficiently lysed the *S. aureus* strain CRISPR spc+ strain (red). The control *S. aureus* CRISPR spc- strain is sensitive to both wild-type phage 812h1 (blue) and phage 812h1His (brown). The *S. aureus* CRISPR spc+ strain is not sensitive to phage 812h1 (green).

The long-read sequencing confirmed the insertion of the *his*-tag sequence into the *tsp* gene, resulting in the rescue of recombinant 812h1His phages from the immune system of the *S. aureus* CRISPR spc+ strain (Figure S2). Sequence analysis showed an 85.7% occurrence of complete (6× CAT) *his*-tag insertion at 1061 × coverage (SD = 0.9). The remaining 14.3% of recombinant phages contained shortened variants of the *his*-tag sequence; however, no uniform sequence subpopulation was identified (Figure S2).

The spatial accessibility of the poly histidine tag on the virion surface was confirmed using an enzyme-linked immunosorbent assay (ELISA) with peroxidase-modified anti-mouse antibody (HRP-anti-mouse), indicating a specific binding of a monoclonal mouse anti-His IgG antibody conjugated with Alexa Fluor 488 (anti-HisAF488) to the phage 812h1His, while absent from the phage 812h1 (Figure 4A). In addition, we tested if the interaction between the poly histidine tag and the His-tag specific antibody can occur in the complex sample of 50% human serum (Figure S3). The signals of phage 812h1His were in all cases higher than the signals of 812h1, which successfully confirmed the specificity of the interaction, even in serum.

**Figure 4:**
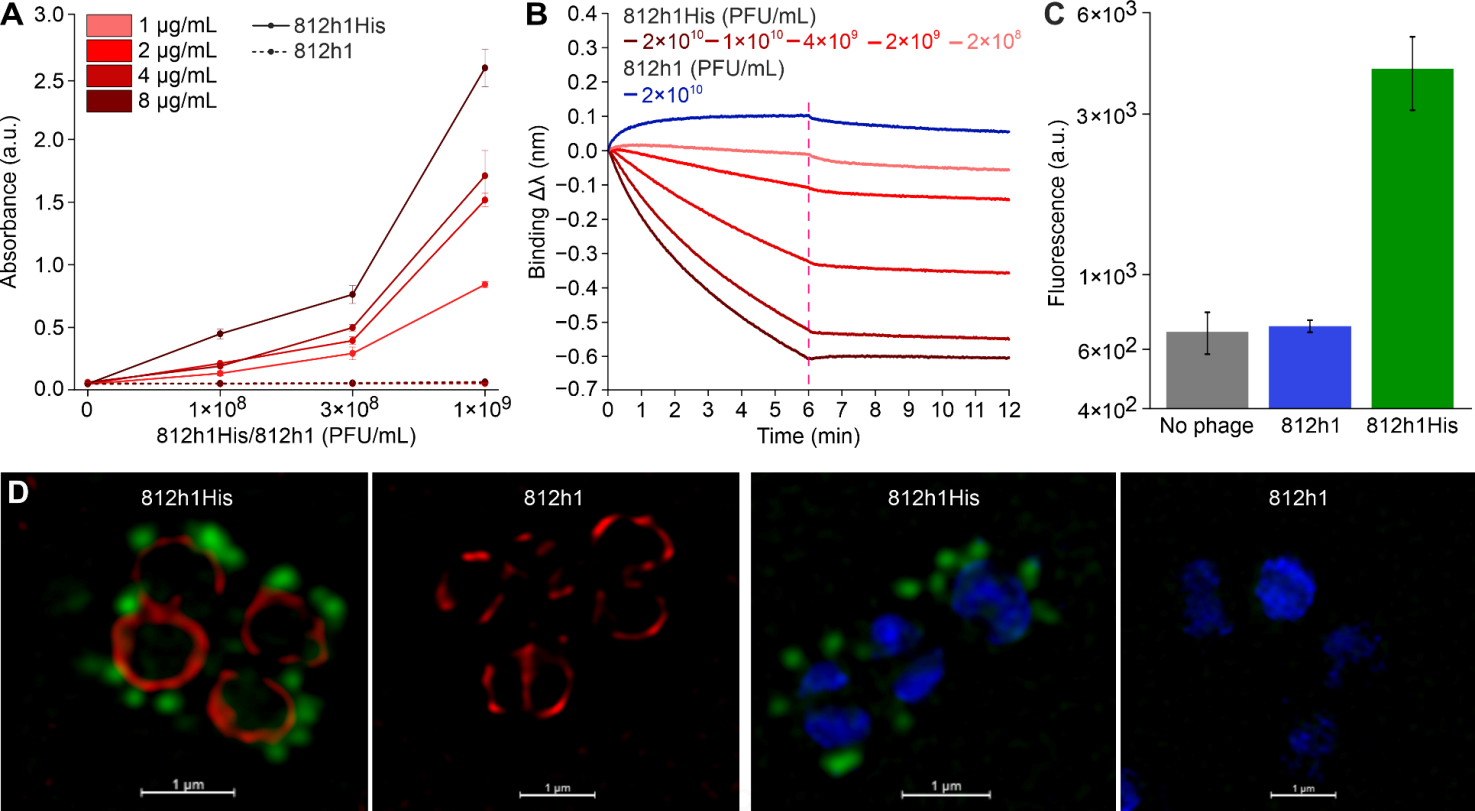
Binding of anti-His antibodies to modified phage 812h1His. (A) ELISA confirmation of the accessibility of the poly histidine tag on phage 812h1His virion surface. A pronounced concentration-dependent binding of the anti-HisAF488 antibody to immobilized 812h1His phage was demonstrated utilizing HRP-anti-mouse antibody as a label. In contrast, binding of the anti-HisAF488 antibody to immobilized 812h1 was not observed. Three immobilization concentrations of phages and four antibody concentrations were tested. (B) Real-time detection of 812h1His phage by Bio-Layer Interferometry. A concentration-dependent binding of the phage 812h1His to the immunosensor was monitored (red color gradient), displaying an inverse binding profile. The dissociation starts in 6 min (red dash line). The control sample of unmodified phage 812h1 displayed low-level nonspecific positive binding (blue). (C) Detection of recombinant phage using flow cytometry determination of the median fluorescence intensity (MFI) exhibited by *S. carnosus* TM300 cells after binding of fluorescently labeled phage 812h1His (green), wild-type phage 812h1 (blue), or no phage (gray). The MFI (812h1His) was significantly elevated (*p* < 0.001) compared to MFI (812h1) as determined by one-way ANOVA and Tukey’s HSD posthoc test. (D) Ultrahigh-resolution fluorescence microscopy of 812h1His–anti-HisAF488 phage-antibody complex (green) adsorbed on the *S. carnosus* TM300 strain, stained by cell membrane-binding SynaptoRed (red) or DNA-binding DAPI (blue) fluorescent dyes. The control samples of wild-type phage 812h1 gave no phage-specific green fluorescence signal. Scale bar, 1 μm.

Next, a biolayer interferometry (BLI) biosensor with an anti-HIS-(HIS2)-functionalized surface was employed for monitoring the binding capabilities of 812h1His to an immunosensor in real-time (Figure 4B). We observed a pronounced concentration-dependent association of 812h1His with the biosensor surface. Due to the relatively large size of the phage particle, which is approximately 330 nm long,^30^ its specific binding to the surface exhibited an inverse (negative) response (Figure 4B), consistent with previous observations for other large complexes and viruses.^38–40^ In contrast, phage 812h1 exhibited a low positive signal response, likely from residual nonspecific binding of small molecules present in the phage sample. Our BLI detection assay thus demonstrates the ability of the immunosensor to distinguish the specific binding of large phage particles from residuals that adsorb nonspecifically. Each modified phage particle contained 312 His-tagged TSP copies,^30^ which are expected to form multivalent interactions with antibodies at the sensor surface. The enhanced overall avidity of the interaction is probably responsible for the observed high binding stability and very low dissociation rate (Figure 4B).

*Staphylococcus carnosus* was used as an organism for immunological detection assays, owing to the absence of protein A in its cell wall, to which the Fc fragments of commonly used commercial anti-His antibodies have high affinity.^41^ Fluorescently labeled 812h1His–anti-HisAF488 phage-antibody complex bound to *S. carnosus* TM300 exhibited a 6-fold increase in median fluorescence intensity measured by flow cytometry compared to the control (Figure 4C). This strong signal enabled the visualization of phage binding using ultrahigh-resolution fluorescence microscopy (Figure 4D). Moreover, the modified bacteriophage tail sheaths were visualized by immunodetection and transmission electron microscopy using 6 nm Colloidal Gold AffiniPure Goat Anti-Mouse IgG secondary antibody (anti-mouse IgG-6 nm gold), which specifically bound to anti-HisAF488 in the phage TSP (Figure 5).

**Figure 5:**
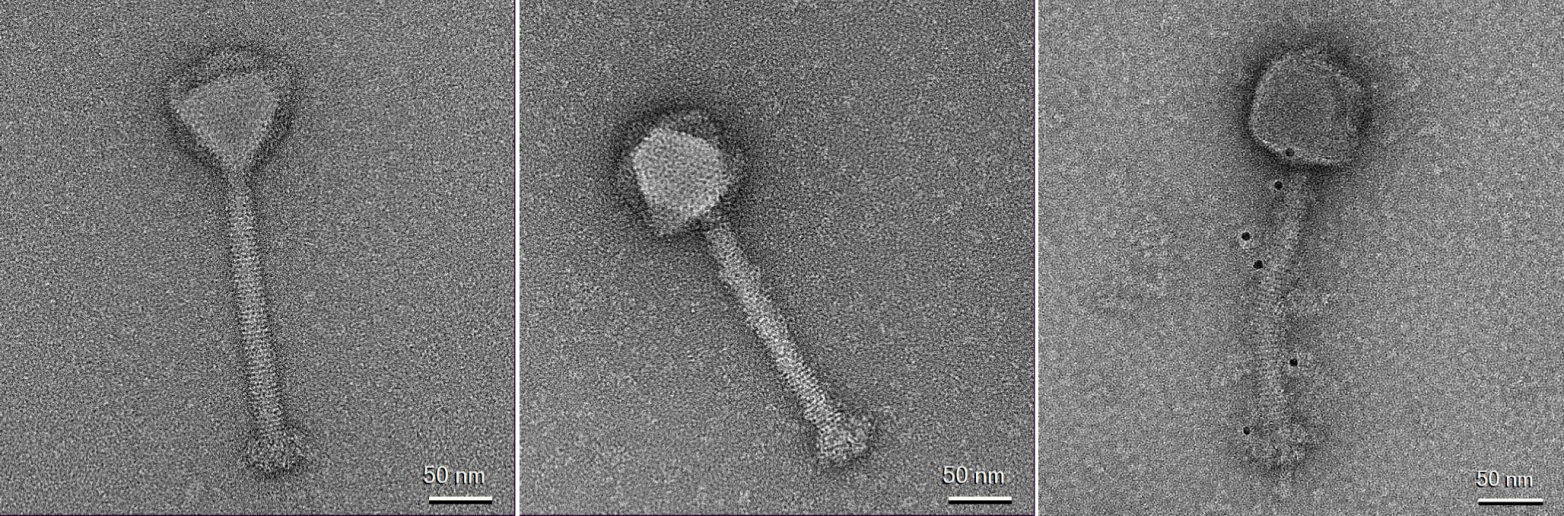
Transmission electron microscopy of phage particles with a monoclonal mouse anti-HisAF488 antibody (from left): wild-type phage 812h1; 812h1His–anti-HisAF488 phage-antibody complex; anti-mouse IgG-6 nm gold secondary antibody binding to 812h1His–anti-HisAF488 phage-antibody complex. Scale bar: 50 nm.

## Conclusions

Labeling phage virion proteins is challenging due to their compact and highly organized structure. Success depends on the structure–function relationship of virion components, making structural data essential for rational engineering. High-resolution structures or reliable models thus help identify suitable insertion sites while minimizing the disruption of essential functions or assembly pathways. Surface-exposed, flexible loops are preferred insertion sites for reporter tags, as they are less likely to affect structural integrity or critical interactions.

This study demonstrates the potential of CRISPR-Cas10-based phage engineering to create customizable structure-guided virion modifications for *Kayvirus* strains. An accessible poly histidine tag incorporated into the phage tail sheath enabled site-specific functionalization of the phage surface, which provides a range of applications, including the immunodetection of viral particles using advanced microscopic techniques, the immobilization of phages on biosensor platforms, and the phage-based flow cytometric detection of bacterial strains.

## Material and Methods

### Bacteria, Bacteriophage, Culture Conditions

*Escherichia coli* TOP10F’ (Invitrogen) was used for cloning. The transformants were cultivated at 37 °C with shaking at 120 rpm in Luria–Bertani (LB) medium (Oxoid) supplemented, if required, with ampicillin (100 μg/mL). Strains for phage editing and modified phage selection were derived from *S. aureus* RN4220.^42,43^ *Staphylococcus carnosus* TM300 ^44^ was used for phage-antibody immunological assays. Staphylococcal strains were cultured at 37 or 30 °C with shaking at 120 rpm in Meat-Peptone Broth (MPB) medium^33^ supplemented, if required, with chloramphenicol (25 μg/mL) and/or erythromycin (10 μg/mL). Bacteriophage 812h1 and its propagation have been described previously.^33^

### DNA and Plasmid Vectors

The plasmid vectors used are given in Table 1. Custom-designed DNA primers and oligonucleotides (Table S1) were purchased from Sigma-Aldrich (Merck). The homology arms segment HR_812HisTSP was synthesized (Twist Bioscience). The cloning and plasmid vector construction workflow using restriction enzymes is described in the Supporting Information.

### Indirect Enzyme-Linked Immunosorbent Assay (ELISA) for His-Phage Testing

Unless stated otherwise, incubations were performed at room temperature for one h with gentle shaking, followed by four washes in washing buffer (50 mM Tris, 0.05% Tween 20, 1 mM KF, pH 7.4). Microlon high-binding 96-well microtiter plate (Greiner Bio-One) was coated with phage 812h1 or 812h1His in coating buffer (100 μL in each well; 50 mM Na_2_CO_3_/NaHCO_3_, 0,05% NaN_3_, pH 9.6) and incubated overnight at 4 °C. Each sample well was treated as follows: blocked with 20% SuperBlock TBS (Thermo Fisher Scientific) in washing buffer, incubated with 6×-His Tag Monoclonal Antibody (HIS.H8) Alexa Fluor 488 (anti-HisAF488) (Invitrogen) at various dilutions (1, 2, 4, and 8 μg/mL in assay buffer consisting of 10% SuperBlock TBS, 0.05% Tween 20, 50 mM Tris, 150 mM NaCl, 1 mM KF, 0.5% PEG, pH 7.5), and incubated with the Peroxidase AffiniPure Goat Anti-Mouse IgG (H+L) (Jackson ImmunoResearch) in assay buffer (0.22 μg/mL). After the final washing, a TMB-Complete2 substrate solution (TestLine Clinical Diagnostics) was added for color development, followed by stopping the reaction with 1 M H_2_SO_4_ and reading absorbance at 450 nm using a Synergy HT microplate reader (Bio-Tek Instruments).

### Bio-Layer Interferometry (BLI) Biosensing

An Octet RED96e system (ForteBio) was employed for a BLI detection binding assay of recombinant phages. All BLI steps were performed in phage buffer at 25 °C. The association and dissociation were monitored for 360 s while being shaken at 1000 rpm in 200 μL of the samples. The phages 812h1 (2 × 10^10^ PFU/mL) or 812h1His (ranging from 2 × 10^8^ to 2 × 10^10^ PFU/mL) diluted in phage buffer and the Octet Anti-HIS (HIS2) precoated biosensors (Sartorius) pre-equilibrated for 120 s in phage buffer were used. The experiment was performed in biological duplicates. Data were collected using Data Analysis v.11.1 software (ForteBio).

### Flow-Cytometry and Fluorescence Microscopy

Bacteriophage 812h1His (at 1 × 10^10^ PFU/mL) was incubated with a 6×-His Tag Monoclonal Antibody (HIS.H8) Alexa Fluor 488 (anti-HisAF488) at a final concentration of 1 μg/mL for 2 h at 4 °C; then, the *S. carnosus* TM300 cell suspension in phage buffer (OD_600 nm_ = 0.6) was added to achieve an input ratio of 10:1 and the mixture was incubated for 10 min. The samples were washed with phage buffer and analyzed with a CytoFLEX S flow cytometer (Beckman Coulter). Three independent biological experiments were performed in a technical triplicate. Data was acquired from 50,000 events per sample and analyzed using the software CytExpert v.2.5 (Beckman Coulter).

For fluorescence microscopy, the staphylococcal cells were stained with DAPI (Thermo Fisher Scientific) or SynaptoRed (Biotium) and washed with phage buffer according to the manufacturer’s recommendations. The stained cells were mixed with fluorescently labeled 812h1His–anti-HisAF488 phage-antibody complex and imaged using high-resolution microscopy (ZEISS Elyra 7 with Lattice SIM).

### Transmission Electron Microscopy and Immunoelectron Microscopy

For the immunoelectron microscopy, the purified bacteriophage 812h1His (at 1 × 10^10^ PFU/mL) was incubated with 6×-His Tag Monoclonal Antibody (HIS.H8) Alexa Fluor 488 (anti-HisAF488) (final concentration 1 μg/mL; Invitrogen) for 2 h at 4 °C, and then 6 nm Colloidal Gold AffiniPureTM Goat Anti-Mouse IgG (Jackson ImmunoResearch) was added according to the manufacturer’s recommendations. All negatively stained samples were prepared by double staining in 2% uranyl acetate. All samples were observed with a Tecnai F20 Transmission Electron Microscope (FEI Company) operated at 200 kV at a magnification of 150,000×.

### Data Visualization and Statistical Analyses

The statistical evaluation, data visualization, and graphing were done using OriginPro 2023 v.10.0 (OriginLab Corporation). Fluorescence microscopy image processing was done using ZEN Lite v.3.11 (Carl Zeiss Microscopy). The structure of the modified tail sheath protein was predicted by AlphaFold2.^46^ Structures were visualized using ChimeraX.^47^

## Supplementary Material



## Data Availability

The sequences of new plasmid constructs and data associated with this work are provided in the Zenodo depository https://doi.org/10.5281/zenodo.15463060. Sequencing data of modified 812h1His phage were deposited in GenBank under the BioProject number PRJNA1268361.

## Notes

Additional experimental details: the structure of the 812h1His tail sheath (Figure S1), sequence analysis of the *tsp* gene region in modified phages 812h1His (Figure S2), detection of modified phages 812h1His in 50% human blood serum by ELISA (Figure S3), primers and oligonucleotides used in this study (Table S1) and Supplementary Methods (PDF)
