# Supplementary material for: CRISPR-Cas10-Assisted Structural Modification of Staphylococcal Kayvirus for Imaging and Biosensing Applications
Source: ACS Synth Biol. 2025 Jul 28;14(8):2979–86. doi: 10.1021/acssynbio.5c00387 (PMC12362608; doi:10.1021/acssynbio.5c00387)
Supplement: Supplementary file 1 [file sb5c00387_si_001.pdf]

SUPPORTING INFORMATION

CRISPR-Cas10-Assisted Structural Modification of Staphylococcal *Kayvirus* for Imaging and Biosensing Applications

Authors

Hana Šimečková[a], Pavol Bárdy[a,b], Lucie Kuntová[a], Eliška Macháčová[c], Tibor Botka[a], Ján Bíňovský[d,e], Josef Houser[d,e], Zdeněk Farka[c], Pavel Plevka[d], Roman Pantůček[a] and Ivana Mašlaňová[a*]

Affiliations

[a]Department of Experimental Biology, Faculty of Science, Masaryk University, Brno 611 37, Czech Republic

[b]Department of Chemistry, York Structural Biology Laboratory, University of York, Heslington, York YO10 5DD, United Kingdom

[c]Department of Biochemistry, Faculty of Science, Masaryk University, Brno 625 00, Czech Republic

[d]Central European Institute of Technology, Masaryk University, Brno 625 00, Czech Republic

[e]National Centre for Biomolecular Research, Faculty of Science, Masaryk University, Brno 625 00, Czech Republic

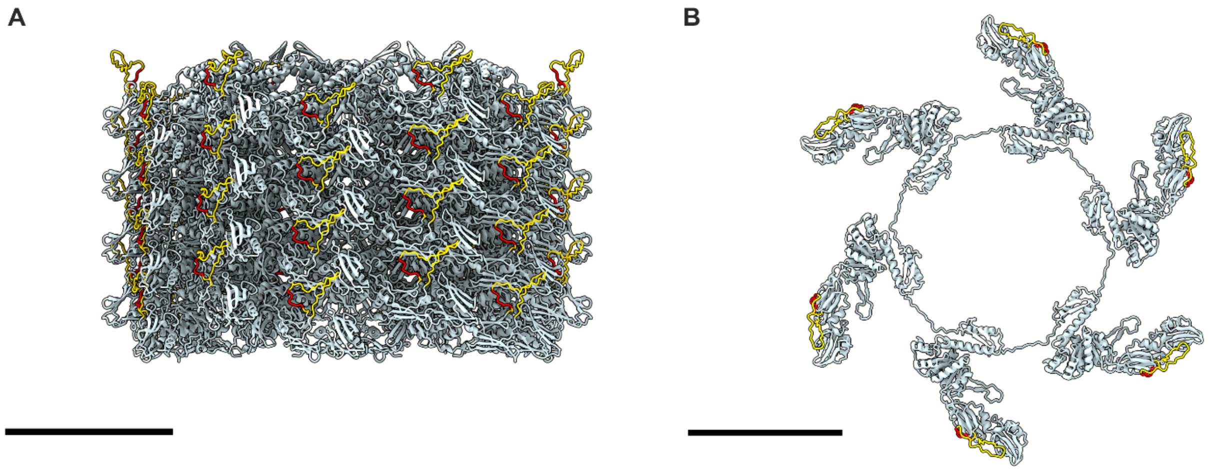

Figure S1. Structures of contracted 812h1His tail sheath. (A) Modified TSP with exposed loop (yellow) and inserted His-tag (red) modeled based on the structure of the four contracted tail sheath discs.[1] Side view of the tail sheath is shown. (B) Top view of a modeled contracted tail sheath disc. The prediction shows a significant flexibility of the exposed loop (yellow, region 271–297) with an embedded His-tag (red) also in the contracted form of TSP. Predictions were made using AlphaFold2. Scale bars are 10 nm (black).

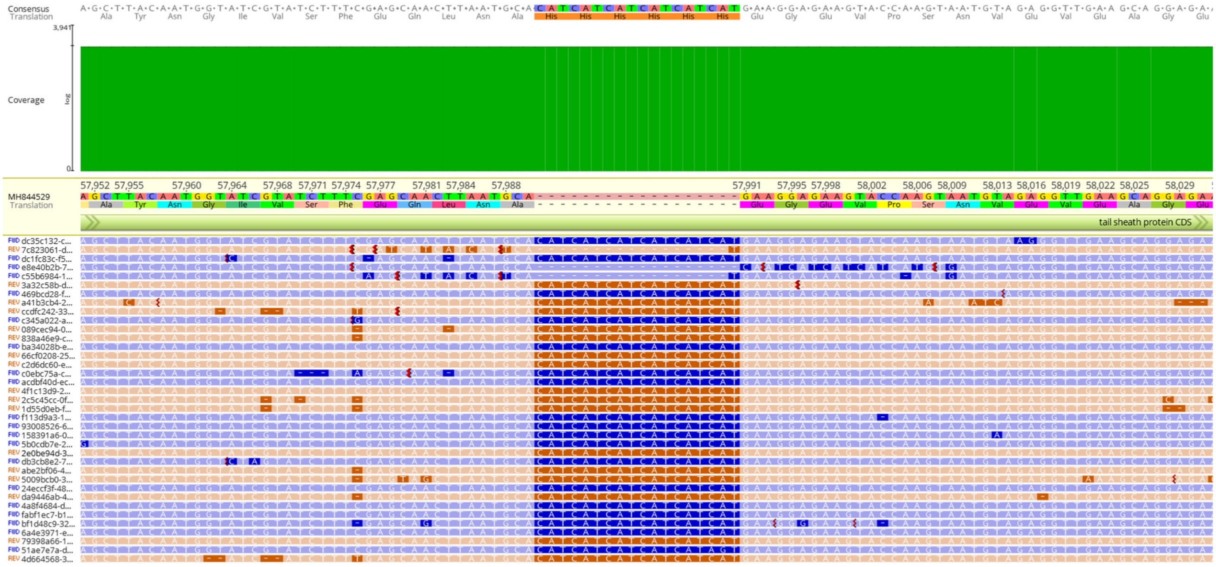

Figure S2. Sequence analysis of the *tsp* gene region in modified phages 812h1His using long-read sequencing. Mapping of ONT sequencing reads of modified phage 812h1His DNA to the wild-type 812h1 genomic sequence (GenBank MH844529) using the Minimap2 plugin v.2.24 (K-mer length 21) in the Geneious Prime v.2025.1.2 program, focusing on the region of the *tsp* gene, confirmed insertion encoding a histidine hexamer (6× CAT). Disagreements with the phage reference sequence are highlighted.

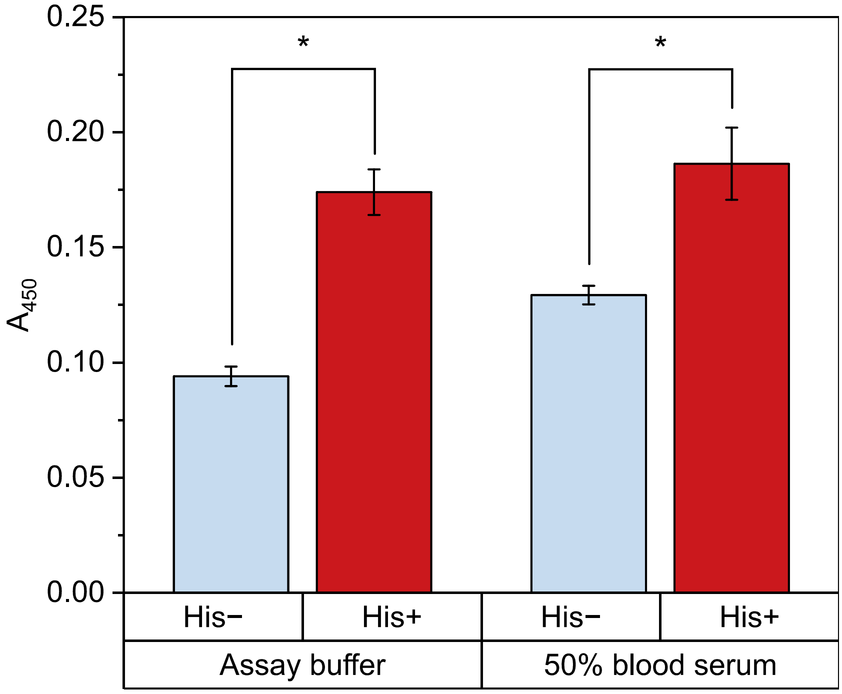

Figure S3. Detection of wild-type 812h1 and 812h1His phages diluted in the complex sample of 50% human blood serum using sandwich ELISA. Wild-type 812h1 phage (His-), 812h1His phage (His+). As coating the microtiter plate with phage is not applicable in the case of complex samples, a sandwich ELISA was designed, based on capture anti-HisAF488 antibody binding the phages, followed by the detection with the Anti-HIS6-Peroxidase monoclonal antibody. The statistical significances ($p < 0.05$) are marked with asterisks.

Table S1. Primers, oligonucleotides, and synthesized dsDNA used in the study

Synthetized DNA	Sequence	Length (nt)	Purpose
gRNA_812TSP_A	5’GTTACCGATCGATACCCACCCCGAAGAAAAGGGGACGAG AACTCTCCTTCTGCATTAAGTTGCTCGAAAGATACGATG	78	pCN_gRNA_812spc+ vector construction: insertion of the cassette containing the spacer into the pCN_gRNAspc- vector
gRNA_812TSP_B	5’AATTCATCGTATCTTTCGAGCAACTTAATGCAGAAGGAGA GTTCTCGTCCCCTTTTCTTCGGGGTGGGTATCGATCG	77	
pHR_mcs_A	5’GATTCTAGAATCCATGGATGCTAGCTAG	28	pHR_812HisTSP vector construction: insertion of the pHR_mcs cassette into the shuttle vector backbone
pHR_mcs_B	5’GATCCTAGCTAGCATCCATGGATTCTAGAATCTGCA	36	
pHR_prim_F	5’GATATCAAAATTATACATGTCAACGATAATAC	32	PCR and sequencing primers for verification of the inserted HR_812HisTSP insertion sequence in vector pHR_812HisTSP
pHR_prim_R	5’ACCGTATTACCGCCTTTGAGTG	22	
HR_812HisTSP	5’ 517 nt …GCACATCATCATCATCATCATGAA… 517 nt	1058	dsDNA segment for homologous recombination: 2× 520 nt homology arms flanking inserted 18 nt His-tag (6× CAT)* underlined

SUPPLEMENTARY METHODS

DNA Cloning Techniques and Reagents. Plasmid DNA was isolated using the NucleoSpin Plasmid kit (Macherey-Nagel) according to the manufacturer's instructions. The DNA concentration was determined spectrophotometrically using the NanoDrop 2000c (Thermo Fisher Scientific). The restriction endonucleases *Bst*EII-HF, *Eco*RI-HF, *Bam*HI-HF, and *Kpn*I-HF (all from New England BioLabs; abbreviated NEB) were utilized for cloning purposes. Digested DNA was purified using the Monarch PCR and DNA Cleanup Kit (NEB), ligated with inserts using the Quick Ligation Kit (NEB), and transformed into competent *E. coli* TOP10F' cells. The constructs were verified by Sanger sequencing using primers pHR_prim_F and pHR_prim_R (Table S1) in the Eurofins MWG Operon sequencing facility (Ebersberg, Germany). The oligonucleotides (Table S1) were annealed in a reaction containing 1 µl of oligonucleotide_A (100 µM), 1 µl of oligonucleotide_B (100 µM), 5 µl T4 DNA ligase buffer (NEB), and 1 µl T4 polynucleotide kinase (10 U/µL, NEB) in 50 µl H_2O; incubated for 1 hour at 37 °C. Subsequently, 2.5 µl of 1 M NaCl was added, and the temperature was increased to 95 °C for 5 minutes. Then the temperature was gradually decreased by 1 °C/min until reaching 25 °C. The annealed oligonucleotides were immediately used for cloning experiments.

Construction of Shuttle Vectors. The cassette containing the P*cap1A* promoter[2] and the *S. epidermidis* CRISPR-Cas10 gRNA scaffold was synthesized and inserted into the pCN51 vector between the restriction sites *Kpn*I and *Eco*RI, leading to the vector pCN_gRNAspc-. Protospacers were selected according to the criteria for the Type III-A CRISPR-Cas10 system: (a) 35 nucleotides in length, (b) within transcribed coding regions, (c) with no complementarity between the Tag sequence (5′-ACGAGAAC region on the gRNA) and the corresponding Antitag sequence adjacent to the protospacer. A protospacer sequence containing the target editing site within the *tsp* gene was designed (5′-ATCGTATCTTTCGAGCAACTTAATGCAGAAGGAGA), overlapping with the region to be modified. The oligonucleotides gRNA_812TSP_A and gRNA_812TSP_B (Table S1) were annealed and cassette gRNA_812TSP inserted into the pCN_gRNAspc- vector (between the restriction sites *Bst*EII and *Eco*RI), resulting in the final vector pCN_gRNAspc+. The backbone of the pCas9counter vector was utilized for constructing the pHR_812HisTSP vector. By restriction digestion with the *Pst*I and *Bam*HI endonucleases, the Cas9 enzyme was removed, and subsequently the multiple cloning site cassette (annealed oligonucleotides pHR_mcs_A and pHR_mcs_B; Table S1) was introduced. Between the *Bam*HI and *Eco*RI restriction sites, the HR_812HisTSP segment for homologous recombination was inserted, resulting in the vector pHR_812HisTSP. The codon for histidine (CAT) was chosen based on the *S. aureus* NCTC 8325 codon usage table.[3]

Purification of Bacteriophages. Phage lysate (50 mL) was filtered using Filtropur S (0.45 µm; Sarstedt) and pelleted by high-speed centrifugation at 64,000 × g and resuspended in 300 µL of phage buffer (50 mM Tris–HCl, 10 mM NaCl, 10 mM $CaCl_2$, pH 8.0). Concentrated phage particles were then ultracentrifuged on Optima XPN-90 (194,000 × g; rotor SW 55 Ti; Beckman Coulter) in the CsCl gradient (1.45 g/mL, 1.50 g/mL, 1.70 g/mL) at 12 °C for 4:00 hours. Residual CsCl was then removed by dialysis using the Float-A-Lyzer G2 Dialysis Device (MWCO 300 kD, Spectrum Laboratories). Bacteriophage enumeration was performed using a double agar overlay plaque assay, as previously described.[4]

Bacteriophage Editing and Selection. The *S. aureus* RN4220 strain was transformed with vectors by electroporation, as described previously.[5] An overnight culture was inoculated into 50 mL of fresh MPB supplemented with chloramphenicol and erythromycin, and incubated with shaking at 30 °C until the OD_{600} reached 0.4. Then, 5 mL of 812 phage lysate (1×10^9 PFU/mL) and 500 µL of $CaCl_2$ (200 mM) were added, and incubation continued with shaking at 30 °C until lysis occurred. The resulting lysate containing recombinant phages was filtered through a 0.45 µm MFMillipore membrane filter (Merck) and stored at 4 °C. Recombinant phages were selected by iterative propagation on the *S. aureus* CRISPR spc+ selection strain. The sensitivity of bacterial strains to phages was determined turbidimetrically using the Multiskan™ FC Microplate Photometer (Thermo Fisher Scientific). An overnight culture was diluted to an OD_{600} of 0.9 (3×10^8 CFU/mL), and 200 µL was aliquoted into the wells of a 96-well plate (Thermo Fisher Scientific). Then, 60 µL of phage lysate (1×10^8 PFU/mL) was added, resulting in a multiplicity of infection (MOI_{input}) of 0.1. The OD_{600} was measured at 37 °C with pulsed shaking every 10 minutes for 14 hours.

Sequencing of Recombinant Phage DNA. Concentrated phage particles in 400 µL of phage buffer (50 mM Tris, 10 mM NaCl, 10 mM $CaCl_2$, pH 8.0) were incubated for 30 min at 37 °C with 1 µL DNase I (1 U/µL; Thermo Fisher Scientific) and 1 µL RNase A (10 mg/mL; NEB). Next, 30 µL of 10% SDS and 10 µL of proteinase K (20 mg/mL; Sigma-Aldrich) were added to the sample and incubated for 15 min at 55 °C. DNA was purified using a Genomic DNA Clean & Concentrator-25 kit (ZymoResearch). The obtained DNA was sequenced on the Oxford Nanopore Technologies platform (SQK-LSK114 kit, FLO-FLG114 flow cell) using the software MinKNOW v.24.11.10, also used for base-calling (Super-accurate model) and trimming. The sequencing reads were mapped to the reference (GenBank: MH844529.1) using the Minimap2 v.2.24 plugin in Geneious Prime v.2025.1.1 (GraphPad Software).

Sandwich ELISA for HIS-Phage Testing in Blood Serum. A 96-well microtiter plate was coated with 6×-His Tag Monoclonal Antibody (HIS.H8), Alexa Fluor 488 (anti-HisAF488) diluted in coating buffer (8 µg/mL, 100 µL/well) and incubated overnight at 4 °C. Next, the following

steps were carried out at room temperature, all incubations were performed for 1 hour under gentle shaking; the plate was washed four times with 250 µl of washing buffer after each step. The microtiter plate was blocked with 200 µL of 20% SB in washing buffer. Then, phage 812 with or without HIS tag was diluted in assay buffer or 50% serum (3×10^8 PFU/mL) and added to each well (100 µL/well) and incubated. Subsequently, the Anti-HIS6-Peroxidase monoclonal antibody (Sigma-Aldrich) in assay buffer (0.5 U/mL) was added to each well (100 µL) and incubated. After the final washing steps, followed the measurement by the addition of 100 µL/well of TMB-Complete2 substrate solution and after color development subsequent stopping of the enzyme reaction using 1 M H_2SO_4 (100 µL/well) and readout at 450 nm (A_{450}) by a microplate reader (Synergy HT, Bio-Tek Instruments).

SUPPLEMENTARY REFERENCES

(1) Bíňovský, J.; Šiborová, M.; Nováček, J.; Bárdy, P.; Baška, R.; Škubník, K.; Botka, T.; Benešík, M.; Pantůček, R.; Tripsianes, K.; et al. Cell Attachment and Tail Contraction of *S. aureus* Phage phi812. *bioRxiv* **2024**, preprint. DOI: 10.1101/2024.09.19.613683

(2) Ouyang, S.; Lee, C. Y. Transcriptional Analysis of Type 1 Capsule Genes in *Staphylococcus aureus*. *Mol. Microbiol.* **1997**, *23*, 473-82. DOI: 10.1046/j.1365-2958.1997.d01-1865.x

(3) Nakamura, Y. Codon Usage Database. Codon usage table [online] https://www.kazusa.or.jp/codon/cgi-bin/showcodon.cgi?species=93061.

(4) Kropinski, A. M.; Mazzocco, A.; Waddell, T. E.; Lingohr, E.; Johnson, R. P. Enumeration of Bacteriophages by Double Agar Overlay Plaque Assay. *Methods Mol. Biol.* **2009**, *501*, 69–76. DOI: 10.1007/978-1-60327-164-6_7

(5) Grosser, M. R.; Richardson, A. R. Method for Preparation and Electroporation of *S. aureus* and *S. epidermidis*. *Methods Mol. Biol.* **2014**; *1373*, 51-57.2. DOI:10.1007/7651_2014_183.
